# Perforated Appendicitis in a Neonate Presenting with Intestinal Obstruction

**Published:** 2013-04-01

**Authors:** Pierrot Sarkis

**Affiliations:** Department of Pediatric Surgery, Saad Specialist Hospital, Al-Khobar- 31952, Saudi Arabia

**Keywords:** Neonate, Perforated appendicitis, Internal hernia, Intestinal obstruction

## Abstract

We report a case of neonatal perforated appendicitis presenting with an early picture of intestinal obstruction secondary to entrapment of small bowel under inflamed appendix vermiformis.

## INTRODUCTION

Acute appendicitis is rare in neonates. Clinical presentation is always variable and non-contributory to the diagnosis. Final diagnosis is usually confirmed during surgery, late in the course which increases the risk of morbidity [1-4]. We report a case of neonatal appendicitis (NA) presenting with intestinal obstruction and presented unusual operative findings.

## CASE REPORT

A 21-day-old male neonate presented with fever, abdominal distension, and vomiting for two days. He had normal perinatal history (born at term with weight of 2.3kg) with normal feeding and thriving trends. On examination, his temperature was 39˚C with peripheral cyanosis and tachycardia. Abdominal examination revealed generalized distension and tenderness. CBC showed leukocytosis (27000/µL) and CRP was 70 mg/dl. Abdominal X-ray showed few air-fluid levels. Ultrasound abdomen was unremarkable except for minimal peritoneal fluid. The patient was managed with general neonatal supportive measures and prophylactic antibiotics. 

Exploratory laparotomy was done on the same day of admission that revealed dilated small bowel loops and the last 20 cm of ileum was entrapped under the appendix, the tip of which was adherent with the mesentery of small bowel (Fig. 1). The appendicular tip had a perforation as well. The bowel beyond entrapment showed signs of compromised blood supply which returned progressively to normal after releasing tip of the appendix from the mesentery. No generalized peritonitis or intra-abdominal free fluid was found. Appendectomy was performed. Patient had uneventful postoperative period and discharged in a good general condition. Histopathology of the appendix showed acute appendicitis with inflammation of the peri-appendicular tissue, with normal ganglion cells. At follow-up he is thriving well with no other complaints.

**Figure:**
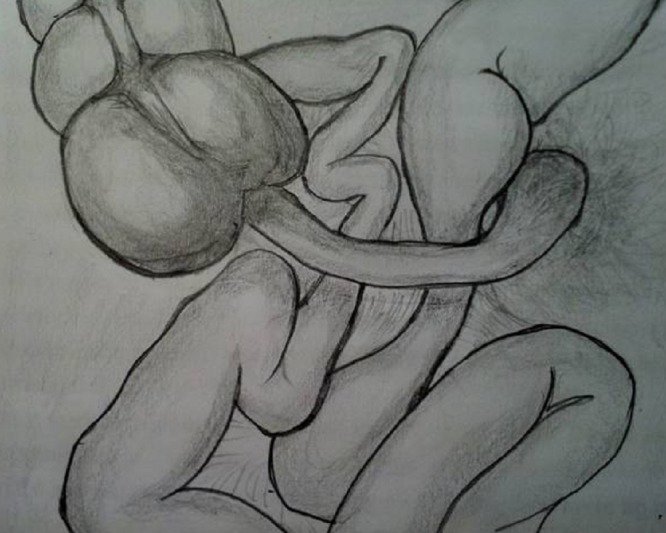
Figure 1: Illustration showing small bowel entrapment by appendix fixed at the tip.

## DISCUSSION

Most authors relate perforation in NA to late presentation and diagnosis [4]. In contrast, the findings in the present case confirm that perforation can occur early in the process of appendicitis as also shown by cases of Khan, Gupta, and Kayastha [1-3]. Intestinal obstruction in acute appendicitis is usually attributed to local abscess or mass formation or generalized peritonitis, or it can be adhesions in late postoperative course [1-5]. In our case, there was no abscess or generalized peritonitis. Surprisingly, the short appendix fixed at its tip was entrapping small bowel through a free space between tip of perforated appendix and small bowel mesentery which led to the presentation with early signs of intestinal obstruction. This condition is scarcely reported in other age groups and termed as appendicular knot or appendicular tie syndrome [6, 7]. To the best of our knowledge, no such case has been reported in neonates.

## Footnotes

**Source of Support:** Nil

**Conflict of Interest:** None

**Editorial Comment:** The terms appendicular knot or appendicular tie syndrome have been attributed to the entrapment of bowel under inflamed appendix adherent at the tip with mesentery or bowel; however, appendicular band syndrome (because appendix when fixed on free end acts as a band; appendicular knot or tie is different in literal meaning) is more appropriate term for this occurrence. 

